# Relationship between the Cumulative Incidence of Kawasaki Disease and the Prevalence of Electrocardiographic Abnormalities in Birth-Year Cohorts

**DOI:** 10.2188/jea.JE20090159

**Published:** 2010-11-05

**Authors:** Kunio Kawai, Mayumi Yashiro, Yosikazu Nakamura, Hiroshi Yanagawa

**Affiliations:** 1 Kouno Clinic, Fukui, Japan; 2 Department of Public Health, Jichi Medical University, Shimotsuke, Tochigi, Japan

**Keywords:** Kawasaki disease, mucocutaneous lymph node syndrome, cumulative incidence, birth-year cohort, prevalence of electrocardiographic abnormalities

## Abstract

**Background:**

Kawasaki disease (KD) causes systemic vasculitis and coronary aneurysms. It frequently results in electrocardiographic (ECG) abnormalities of short duration. Cardiac sequelae persist beyond the acute stage in a few patients. There are many areas to be investigated concerning the effects on the vascular system of patients suffering from KD and its sequelae.

**Methods:**

The cumulative incidences of KD and its cardiac sequelae were calculated in birth-year cohorts, using data obtained from KD nationwide surveys. The results were compared with the prevalence of ECG abnormalities detected in cardiac examinations conducted at primary and secondary schools for each birth-year cohort. This comparison allowed observation of relationships in these trends for each birth-year cohort.

**Results:**

The cumulative incidence of late-stage cardiac sequelae gradually declined. However, there were increases in the cumulative incidence of ECG abnormalities and in the cumulative incidences of KD and acute-stage cardiac disorders related to KD.

**Conclusions:**

The results suggest that even among children without late cardiac sequelae, KD can have a persistent effect on the cardiovascular system. It thus appears necessary to extend clinical observation of children with a history of KD, even if they developed only acute-stage cardiac lesions.

## INTRODUCTION

Since the first report on Kawasaki disease (KD), in 1967, there have been 19 nationwide surveys conducted in Japan from 1970 through 2006.^1^ These biennial retrospective incidence surveys investigated patients with KD who visited target hospitals for the first time. The medical facilities that were requested to participate in the survey were hospitals with pediatric departments and 100 or more beds, or those specializing in pediatrics but with fewer than 100 beds.

The 19th nationwide survey of Kawasaki disease investigated the period from January 2005 through December 2006. Of the 2183 hospitals that were requested to participate, 1543 reported 20 475 patients with KD during this time. The percentage of KD patients recorded in these surveys was reported to exceed 80% of all KD patients.^2^

From the early days of these surveys, vasculitis-related cardiac sequelae, such as coronary aneurysms, were noted, and electrocardiographic (ECG) abnormalities attracted attention. It was found that 72% of KD patients developed ECG abnormalities, although some only temporarily, according to Asai et al.^3^ Although ECG abnormalities are not investigated in the nationwide surveys of KD, the surveys do request a list of patients who received a diagnosis of KD, together with their cardiac sequelae—such as coronary aneurysm, giant coronary aneurysm, coronary dilatation, coronary stenosis, valvular dysfunction, and myocardial infarctions.

In these nationwide surveys, the annual number of KD patients per 100 000 children aged 0 to 4 years is reported. These data are useful for pediatricians who wish to observe the annual epidemiologic trends in KD.^4^^,^^5^ However, it is important that general physicians who perform cardiac examinations in primary and secondary schools know the numbers of students and children who suffer from cardiac sequelae due to KD within a single school grade. For this purpose, reports on annual incidence are not informative enough to identify cardiac sequelae several years after onset. Previous epidemiologic studies indicated that the age-specific incidence of KD peaks at approximately age 1 year. Thus, several years will elapse before the child reaches school age. Cumulative data on the incidence of KD are necessary for general physicians. For internists, it is more important to know the trend in KD incidence for the patient’s age-specific cohort.

We propose a new approach. Using data collected in the nationwide surveys on KD, KD patients were grouped by year of birth, ie, birth-year cohort. The cumulative incidences of KD, acute-stage cardiac lesions, and cardiac sequelae were calculated. Studies have reported the cumulative incidence of KD for each birth-year cohort^6^ and the cumulative incidences of patients with acute-stage cardiac lesions and cardiac sequelae due to KD in each birth-year cohort.^7^

Using these data, we examined the relationship between the cumulative incidence of KD in each birth-year cohort and the prevalence of abnormalities found during school cardiac examinations among these cohorts.

## METHODS

The subjects of the current study were selected from those reported to the 8th to 19th nationwide surveys of KD, which investigated the period from January 1983 through December 2006. They were 153 718 patients who presented for KD treatment for the first time; thus, patients suffering from recurrences were excluded. Those with no record of cardiac complications were assumed to be free of such sequelae.

Starting with the 8th nationwide survey (July 1982 through December 1984), a new question related to cardiac sequelae was added to the survey. Beginning with the 15th nationwide survey (January 1997 through December 1998), the survey sheet was designed in such a manner that sequelae could be recorded on 1 of 2 forms: one for cardiac abnormalities that developed during the early stage (within 1 month) of the disease, referred to as acute-stage cardiac lesions, and the other for detectable persistent cardiac abnormalities (after the first month), referred to as late-stage cardiac sequelae.

**Figure 1.**
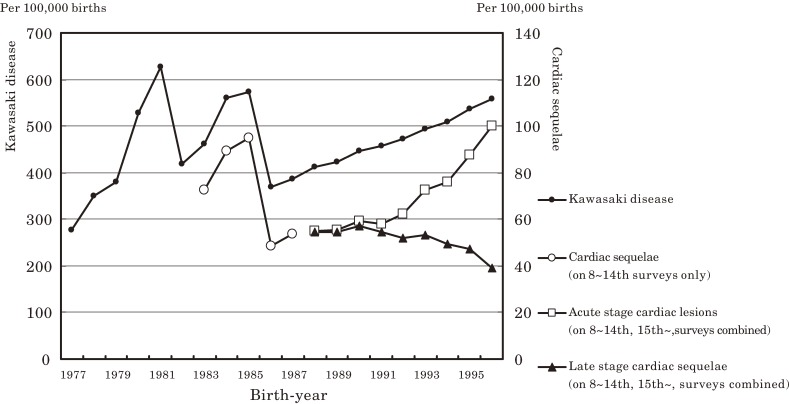
Cumulative incidences of Kawasaki disease and cardiac sequelae at age 10 years, by patient birth year. The cumulative incidences of acute cardiac lesions and late-stage cardiac sequelae were calculated by combining data from the 8th to 14th nationwide surveys with those from the 15th and later nationwide surveys.

In our study of the cumulative incidence of KD in birth-year cohorts, patients with KD were classified by their birth year, and the cumulative number of patients aged 0 to 9 years for the previous 10 years was expressed as the number per 100 000 individuals born in the respective year. The cumulative number of 0-year patients is the number of those who had KD during the period from January through December of that birth year; therefore, the cumulative number of patients for 10 years represents the number of those with KD from January of their birth year through December of the year when the patients were age 9 years. Similarly, the cumulative incidence of those with cardiac sequelae from KD in each birth-year cohort was expressed as the 10-year cumulative incidence of those having either acute- or late-stage lesions due to KD between the ages of 0 and 9 years. Thus, those identified as having cardiac sequelae in one of the items included on the 8th through 14th surveys (time of observation, January 1983 through December 1996) were classified as having cardiac lesions in both the acute and late stages, as shown in Figure 1.

Cardiac sequelae were covered by one item on the questionnaire included in the 8th through 14th nationwide surveys. The subject of this item could be regarded as late-stage lesions, which are investigated in an item added to the questionnaire for the 15th survey. However, after the change of questionnaire, the reported number of patients with late-stage lesions due to KD suddenly dropped in the 15th survey, as compared with the number of the patients with such lesions reported in the 8th through 14th surveys. This indicates that the numbers for patients with cardiac sequelae in the 8th through 14th surveys included those with the acute-stage lesions. Therefore, in the 8th through 14th surveys, the number of patients with “cardiac sequelae” was greater than the number of those with late-stage lesions and lower than the number of patients with acute-stage lesions. It is necessary to accumulate data for several years to calculate the cumulative incidence of KD.

Those listed with “cardiac sequelae” in one of the items included in the 8th through 14th surveys were counted as having both acute- and late-stage cardiac lesions, and in the cohorts from 1988 to 1996, provisional cumulative incidence rates of acute-stage cardiac lesions and late-stage cardiac sequelae of KD in the birth-year cohort were calculated.

These data on KD were compared with those obtained from the annual Report of the Statistical Survey on School Health published by the Ministry of Education, Culture, Sports, Science,^8^ which categorizes its results by student birth year. In the Statistical Survey on School Health, there are data on the prevalence of cardiac diseases and cardiac anomalies, hereafter referred to as the prevalence of cardiac diseases. The prevalence of ECG abnormalities is also reported. The prevalence of cardiac diseases and the frequency of ECG abnormalities revealed by school cardiac examinations within a single school grade can be characterized as the prevalence of abnormalities among a birth-year cohort of pupils. These comparative studies were conducted to identify changes in birth-year cohorts by observing the cumulative incidence of KD and the prevalence of cardiac diseases and ECG abnormalities in related examinations.

The prevalence of cardiac disease and cardiac anomalies was surveyed for each school year. Starting in 1995, ECG examinations were given to children aged 6, 12, and 15 years, which corresponds to entry into elementary, junior high school, and senior high school, respectively. The proportions of those who had cardiac diseases for each school year and the proportions of ECG abnormalities found at ages 6 and 12 were converted to percentages or number per 100 000 for each birth-year cohort. Secular trends were analyzed by comparing them against those for the cumulative incidence of KD for each birth-year cohort, which were obtained from the nationwide surveys.

Next, we focused on ECG examinations given at age 6, and the graphs of the secular trend in the prevalence of cardiac diseases and ECG abnormalities observed in cardiac examinations at schools were compared against the cumulative incidence of acute-stage cardiac lesions and cardiac sequelae due to KD, obtained from the nationwide surveys, in which the incidences were listed for each birth-year cohort. The cumulative number of patients with cardiac lesions at age 6 years was calculated for each patient birth-year cohort by totaling the number of those suffering from cardiac sequelae due to KD between January of their birth year and December of the year when they became 5 years of age.

## RESULTS

Figure 1 shows graphs of the cumulative incidence of KD per 100 000 births and the cumulative incidences of cardiac lesions and late-stage cardiac sequelae at age 10 years for each birth-year cohort, according to the nationwide surveys. The secular trend for the cumulative incidence of cardiac sequelae for birth-year cohorts before 1990 parallels that for the cumulative incidence of KD. After 1991, there were rises in both the cumulative incidences of acute-stage cardiac lesions and KD; however, the cumulative incidence of late-stage cardiac sequelae gradually declined after 1991.

**Figure 2.**
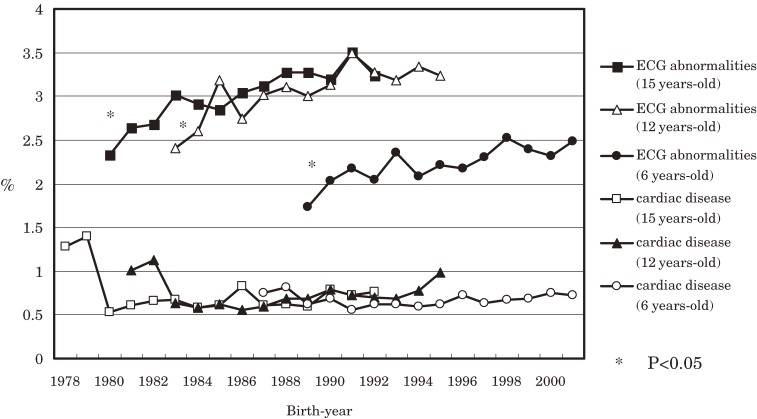
Percentages of cardiac disease abnormalities on electrocardiography (ECG), by birth-year cohort, among students aged 6, 12, and 15 years (data obtained from the Statistical Survey on School Health). Linear regression analysis was conducted to investigate the statistical significance of the observed trends.

Figure 2 shows the percentages of cardiac diseases and ECG abnormalities detected at school health examinations at age 6, 12, and 15 years, adjusted for birth-year cohort. The prevalence of ECG abnormalities was higher at ages 12 and 15 than at 6, while prevalences at ages 12 and 15 were generally similar. When prevalence was compared by birth year, a general increase was noted. Linear regression analysis revealed that the frequency of ECG abnormalities, adjusted for birth-year cohort, increased significantly (*P* < 0.05). In contrast, the prevalence of cardiac diseases showed few age-related differences at ages 6, 12, and 15 years. Sequential comparisons by birth year also revealed a generally stable pattern, with deviations limited to 0.5% to 1%. There was no statistically significant trend in the prevalence of cardiac diseases adjusted for birth-year cohort.

**Figure 3.**
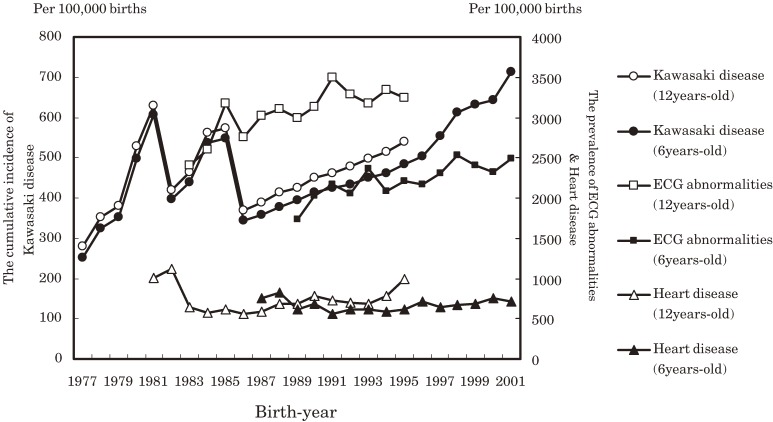
Cumulative incidence of Kawasaki disease and the prevalences of cardiac disease and abnormalities on electrocardiography (ECG), by birth-year cohort, among students aged 6 and 12 years.

Figure 3 shows the secular trend in the prevalence of ECG abnormalities and cardiac diseases detected at school health examinations in children aged 6 and 12 years compared with the cumulative incidence of KD obtained from the nationwide surveys. Examined sequentially by birth year, the graph for the prevalence of ECG abnormalities gradual increased in a manner similar to that for the cumulative incidence of KD. At age 12, the change faithfully reflected the sudden dramatic increase in the number of KD patients born in 1985. In addition, among the cohorts from the years 1986 to 1995, the slope of the curve for the prevalence of ECG abnormalities is similar to that for the cumulative incidence of KD. However, after 1995, there was a sharp increase in the cumulative incidence of KD at age 6, whereas the prevalence of ECG abnormalities only slightly increased. The change in the prevalence of cardiac diseases did not parallel the trend in the cumulative incidence of KD.

**Figure 4.**
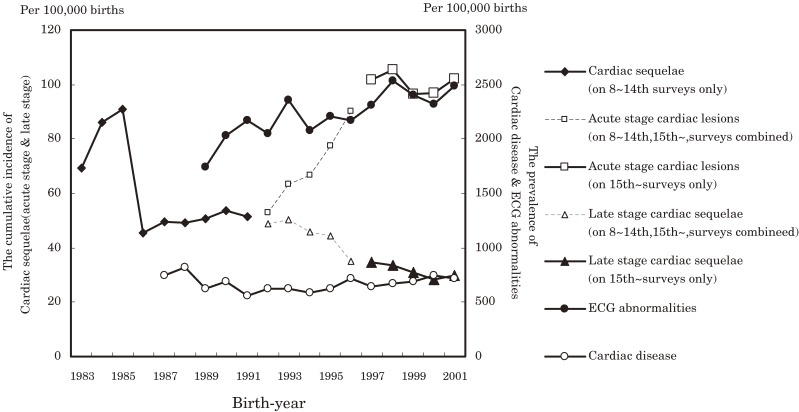
Cumulative incidence of cardiac sequelae of Kawasaki disease and the prevalences of cardiac disease and abnormalities on electrocardiography (ECG), by birth-year cohort, among students aged 6 years. The cumulative incidences of acute cardiac lesions and late-stage cardiac sequelae were calculated by combining data from the 8th to 14th nationwide surveys with those from the 15th and later nationwide surveys.

The graphs for the cumulative incidences of acute-stage cardiac lesions and late-stage cardiac sequelae due to KD for the birth-year cohort at age 6 (from nationwide surveys) were compared with those for the prevalence of ECG abnormalities and heart diseases detected at school health examinations (Figure 4). Since 1997, the changes in the cumulative incidence of acute-stage cardiac lesions were accurately reflected in the occurrence of ECG abnormalities.

The graph for the development of cardiac diseases was not similar to those for the cumulative incidence of acute-stage cardiac lesions or those for cardiac sequelae.

## DISCUSSION

The data on the prevalence of ECG abnormalities and cardiac diseases in the current study were gathered from the annual Report of the Statistical Survey on School Health (1993–2007).^6^ The report is prepared by extracting samples, rather than by studying the entire student population. In the latest report, the 2007 Statistical Survey on School Health, the data on students were extracted from 22.4% of Japanese students aged 5 to 17 years. In the process of sample extraction, schools are first stratified by prefecture or other legislative district, type of school, and number of students. Within each group thus stratified, simple randomization was used to determine the schools at which a survey was to be conducted. The rates of occurrence of cardiac diseases and anomalies and ECG abnormalities are reported annually. It can be assumed that these rates generally agree with overall incidences among Japanese students. If one posits that the students in a single grade level constitute a population group all born within a single year, it is plausible that the occurrence of a cardiac abnormality among the first-grade students, ie, children aged 6 years, may be replaced by the occurrence of a cardiac abnormality developing 6 years later in the cohort of individuals who were born 6 years earlier. The data on the occurrence of diseases thus obtained by adjusting for each birth-year cohort were studied by comparing the cumulative incidence of KD and related cardiac sequelae, both for acute- and late-stage lesions, of a birth-year cohort that had been reported earlier.

The cardiac examination at elementary schools is carried out from April through June for first-graders, when they are 6 years of age. Therefore, the cumulative incidence was calculated by using data until December of the year when the patients were 5 years of age.

Secular trends in the prevalence of cardiac diseases for birth-year cohorts detected at school health examinations show a relative lack of change at ages 6, 12, and 15 years and trivial increases among cohorts. This feature of the prevalence of cardiac diseases appears to differ from that of the cumulative incidence of KD and its acute-stage cardiac lesions and cardiac sequelae in data obtained from the nationwide surveys. However, the graphs for the prevalence of ECG abnormalities in birth-year cohorts that were found in school cardiac examinations show changes similar to those for the cumulative incidence of KD. According to the annual incidence reports of nationwide surveys, there were 3 epidemics of KD, in 1979, 1982, and 1986. Moreover, we previously reported that rises in the cumulative incidence of KD for each birth-year cohort were noted among the birth-year cohort 1 year before the years of these 3 outbreaks. If one examines the data for age 12 years, the prevalence of ECG abnormalities in each birth-year cohort appears to increase, which corresponds to the rising trend for the third outbreak in the 1985 cohort. For the data for the 1986 to 1995 cohort, like the graph for the cumulative incidence of KD, the prevalence of ECG abnormalities at ages 6 and 12 years in the birth-year cohorts has been increasing. In the graphs for the prevalence of ECG abnormalities at age 6 and the cumulative incidence of KD after the 1996 cohort, the patterns are different. However, they both showed a tendency to increase.

In comparison with the graph for the cumulative incidence of acute cardiac lesions at age 6 (according to data from the nationwide survey), the prevalence of ECG abnormalities found during school cardiac examinations exhibited changes similar to those for cohorts dated 1997 or later. This similarity is noteworthy. As stated before, for those cohorts for 1996 or earlier, the method for calculating the cumulative morbidity of acute cardiac lesions was modified because of a change in the questions posed in the nationwide survey. KD patients who would have been classified with cardiac sequelae in the previous survey were now classified as having acute cardiac lesions. Therefore, the numbers for the cumulative incidence of acute-stage lesions before the 1996 cohort should be regarded as provisional. After 1997, however, the figures for the cumulative incidence included only those patients with acute-stage cardiac lesions due to KD, based on results after the 15th survey. Therefore, we may safely assume that patterns are similar for the prevalence of ECG abnormalities detected at school health examinations and the cumulative incidence of acute cardiac lesions obtained from nationwide surveys.

The cumulative incidence of late-stage cardiac sequelae has continued to decline, which differs from the secular trends of the 2 abovementioned graphs.

The current study was limited to a comparison of the cumulative incidence of KD among birth-year cohorts obtained from nationwide surveys and the prevalence of ECG abnormalities calculated from the results of the Statistical Survey on School Health. This study is not analytic; rather, it uses a descriptive approach in which statistical tests are not used in comparing the cumulative incidence of KD with the prevalence of ECG abnormalities. In addition, the nationwide surveys of KD were based on the general population, not selected samples.

We have come to realize that a case-control study between a group of KD patients and a normal group is needed. Hirata et al investigated students who had a cardiac examination during their first year of junior high school, at age 12 years, in Tochigi Prefecture.^9^ In their comparison of children with a history of KD and normal individuals, the prevalence of ECG abnormalities was significantly higher in the former. They also noted that 57% of ECG abnormalities in the former were due to an incomplete right bundle-branch block and right axial deviation. The prevalence of ECG abnormalities was significantly higher in the KD patients, a finding which parallels the data presented in this study. Naturally, there are many causes for ECG abnormalities: they are not limited to KD, but may also be caused by ischemic disease or myocarditis. However, because the sample size in nationwide surveys of KD is large, it is likely that the similarity in the secular trend of the 2 graphs indicates that there is some relationship between them.

Asai et al^3^ investigated the clinical course of KD and found that ECG abnormalities occur at a high rate among patients with KD. In the nationwide surveys, those patients with acute-stage cardiac lesions or cardiac sequelae are equivalent to those who developed coronary artery aneurysm or giant coronary aneurysm, coronary dilatation, coronary stenosis, valvular dysfunction, or myocardial infarction, all of which were due to KD. Therefore, there were more patients with a temporary ECG abnormality than there were KD patients with acute-stage cardiac lesions in those surveys. For example, in the 2001 cohort at age 6 years the cumulative incidence of KD was 713 per 100 000 births and that of acute-stage cardiac lesions was 102.2. The prevalence of ECG abnormalities was 2490 per 100 000 births. The prevalence of congenital heart disease in Japan was reported to be 0.8% to 1.0% per live birth.^10^^,^^11^ The cumulative incidence of KD is up to one half that of ECG abnormalities acquired after birth. Thus, particular attention should be paid to KD patients with temporary ECG abnormalities.

Before embarking on a case-control study, it would be useful to utilize the national survey to ascertain if there is a correlation between the cumulative incidence of KD-related pathologies in each birth-year cohort and the morbidities from other diseases stratified by age.

The first report on KD patients was published approximately 48 years ago. Thus, a number of KD patients have reached adulthood. They consult with internists rather than pediatricians. Therefore, important statistical information on KD should also be provided to general practitioners.

In the current study, fluctuations in the graph for the prevalence of ECG abnormalities at age 6 closely resembled changes in the cumulative morbidity of acute-stage cardiac lesions after 1997. Moreover, the secular trend seen in the graph for the prevalence of ECG abnormalities at age 12 was consistent with the trend for KD. This suggests that KD has a sustained impact on the cardiovascular system, not only in those few KD patients with cardiac sequelae but also in those without evident cardiac dysfunction.

Long-term careful follow-up of KD patients is necessary, not only in those with cardiac sequelae, but also in those who developed only acute-stage cardiac lesions and no cardiac sequelae.
